# Research and Development of Self-Contained Water Injection Systems

**DOI:** 10.3390/ijerph18105392

**Published:** 2021-05-18

**Authors:** Jiri Bazala, Guillaume Hébert, Oliver Fischer, Jürgen Nothbaum, Matthias Thewes, Tobias Voßhall, Peter Diehl, Pavel Kučera

**Affiliations:** 1 Hanon Systems Autopal Services s.r.o., 687 25 Hluk, Czech Republic or Jiri.Bazala@vut.cz (J.B.); ghebert4@hanonsystems.com (G.H.); 2 Institute of Automotive Engineering, Brno University of Technology, Technická 2896/2, 616 69 Brno, Czech Republic; 3 Hanon Systems Deutschland GmbH, 50170 Kerpen, Germany; ofische3@hanonsystems.com (O.F.); jnothbau@hanonsystems.com (J.N.); 4 FEV Europe GmbH, 52074 Aachen, Germany; Thewes@fev.com (M.T.); vosshall@fev.com (T.V.); 5 Consultant, 50667 Köln, Germany; pdiehl@posteo.de

**Keywords:** water injection, compression ratio, self-contained tank, EGR, exhaust condensate

## Abstract

Reducing fuel consumption and thus CO_2_ emissions is one of the most urgent tasks of current research in the field of internal combustion engines. Water Injection has proven its benefits to increase power or optimize fuel consumption of passenger cars. This technology enables knock mitigation to either increase the engine power output or raise the compression ratio and efficiency while enabling λ = 1 operation in the complete engine map to meet future emission targets. Current systems have limited container capacity. It is necessary to refill the water tank regularly. This also means that we cannot get the benefits of an engine with a higher compression ratio. For this reason, the self-contained system was investigated. This article is a methodology for finding the right design of a self-contained water injection system, but also a vehicle test that proves the function.

## 1. Introduction

The need to further reduce fossil fuel consumption in the context of current and future global CO_2_ emission limits requires intensive search for new solutions for automotive engines. For reciprocating internal combustion engines, the mass of CO_2_ emitted into the atmosphere is a function of their fuel consumption. Therefore, research into internal combustion engines is currently focused both on reducing the passive resistances of all mechanisms and on improving the efficiency of their thermodynamic cycles. As for the second option, the most promising solution is to lower the in-cylinder temperature and ensure stoichiometric combustion throughout the engine operating map. The maximum operating conditions of gasoline internal combustion engines are, in general, restricted by the temperature limit of engine components and knocking conditions. Knocking is sharp sound effects caused by premature combustion of part of the com-pressed air-fuel mixture in the cylinder. This phenomenon is destructive for engine itself and it is mainly caused by high temperature of combustion mixture. Knocking is controlled by engine management by fuel enrichment. With modern turbocharged gasoline engines, the maximum acceptable exhaust gas temperature is limited by the thermal material resistance of the turbine. To protect critical components, fuel enrichment (λ < 1) has been used under these conditions. The high vaporization enthalpy of the gasoline enables a significant reduction of the exhaust gas temperature without putting additional thermal load on the cooling system [1].

Nowadays, mixture enrichment is undesired along with the expected extension of regulations in future legislation, which may also include restrictions on Fuel Consumption/CO_2_ emissions. The new emission regulation will require stoichiometric operation (λ = 1) under all engine operating conditions. Consequently, a different medium with a high vaporization enthalpy is required. Based on the patent of Pierre Hugon in 1865 [2], Water Injection (WI) into the combustion chamber of a gasoline engine can also be used to control the temperature of engine components.

Water Injection can be used either for:

- Engine performance improvement or
- Improved fuel consumption

**Figure 1:**
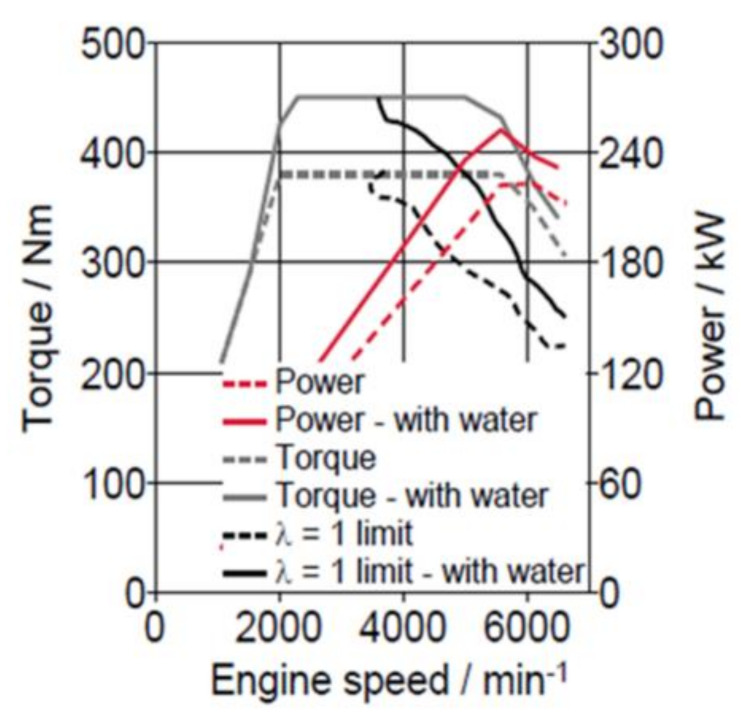
Performance/Torque improvement using water injection [3].

For improved performance, the injection of water into the cylinder lowers the gas temperature, mitigating knocking and allowing a higher load at λ = 1. As shown in Figure 1 below, this increases power/torque characteristics.

**Figure 2:**
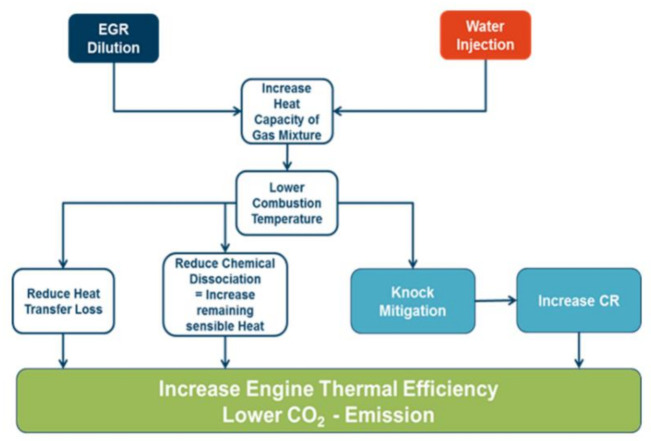
Summarizes the respective effects of EGR and Water Injection.

As far as fuel consumption (FC) improvement is concerned, using WI on a down-sized, turbocharged gasoline engine allows improved combustion phasing and knock mitigation at an increased Compression Ratio (CR) while avoiding fuel enrichment. This will allow stoichiometric operation throughout the entire engine map. Current engine developments seem to concentrate on the effect of “Performance Improvement”, but it can be expected that the development of engines for the mid 2020’s will shift focus to improving fuel consumption [4,5,6,7,8,9]. What both strategies have in common is the use of vaporization enthalpy of a liquid. Injecting Water for vaporization offers an improved cooling effect compared to fuel by a factor of more than 5. It must be mentioned that the “Water Injection”—Technology is only one option of FC improvement through mixture dilution. It competes with Exhaust Gas Recirculation (EGR) in some modes for the same purpose (Figure 2). It has been demonstrated that at medium load a 40–50% Water-to-Fuel Ratio (WFR) with Port Water Injection (PWI) has the same effect as an EGR-rate of 10% [10].

However, WI does have benefits when compared to EGR, especially better controllability as this is not a closed-loop as with EGR, the timing of injection is not linked to other parameters such as turbo charger backpressure, limited inertia (PWI timing not linked to engine operation) and combustion delay (as present with EGR). Additionally, it does not deteriorate combustion stability significantly. The combustion delay linked to EGR dilution and the necessary adaption of the recirculated gas mass flow to the maximum turbocharger characteristics are typically two limiting parameters of the maximum acceptable EGR rate.

## 2. Motivation

**Figure 3:**
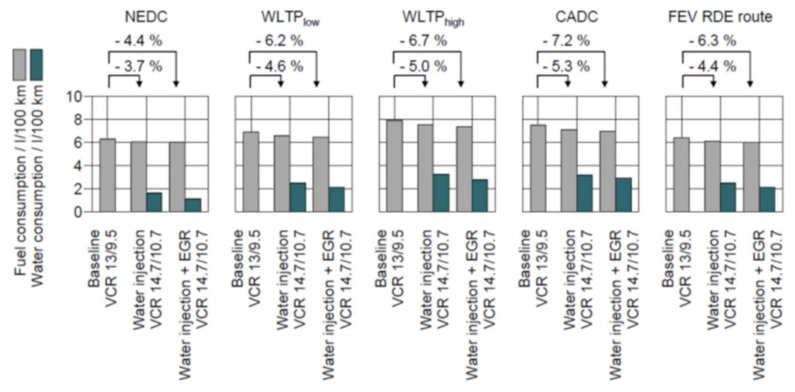
Fuel consumption benefits of EGR and WI for various drive cycles.

As Figure 3 shows, WI has significant effect on fuel consumption. It is without doubt that Fuel Consumption is even lower with a higher compression ratio. Unfortunately, current WI systems in series production are not able to use this maximum possible benefit to their advantage. If the water injection liquid were drained from the tank and the combustion mixture were not cooled through water evaporation, fuel consumption would significantly increase, as evaporation of fuel would take place instead of evaporation of water. To ensure the system has a sufficient amount of water injection liquid, a self-contained tank is necessary.

### Competing on-Board Water Sources

There are limited sources of liquid that can be contained without human refill. These are:

- Harvesting air humidity from ambient (e.g., by A/C condensate)
- Surface Water (e.g., rain water collected from vehicle body)
- Exhaust Gas Condensate

The first two variants are highly dependent upon weather ambient conditions with sufficiently high humidity levels or driver habits (A/C operation is undesirable). Consequently, an adequate supply of water cannot be ensured. On the contrary, the condensation of water vapour formed during gasoline combustion is a reliable source of water. The temperature and humidity levels have only a minor contribution to the full amount of water being present in the exhaust gas. Almost all water in exhaust comes from a combustion reaction from carbohydrates and oxygen from air, not from humidity in air. This can be seen in Equation (1) where ideal combustion is described.

(1) $$C_{8}H_{18}+12.5\left(O_{2}+3.76N_{2}\right)\to \;8CO_{2}+9H_{2}O+47N_{2},$$

The formula above can calculate that 1 kg of fuel on the left side of the formula is 1.4 kg of water vapour on the right side which can be harvested as liquid for WI.

## 3. WAHASY Efficiency

**Figure 4:**
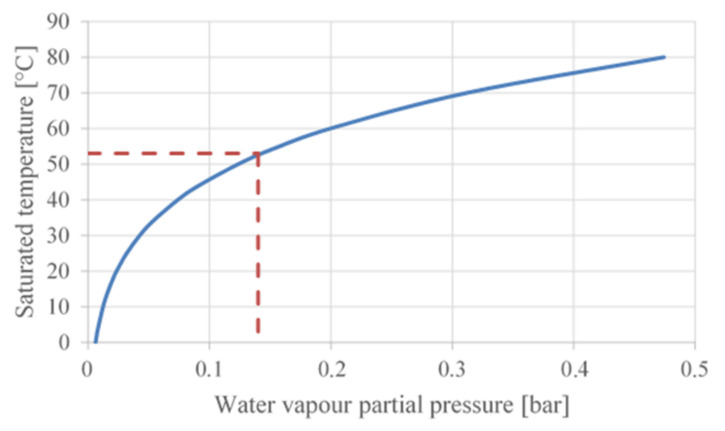
Water saturation pressure.

The fact that water vapour (WC) is present in exhaust is already known. In order to harvest water from exhaust, it is necessary to condensate water vapour to water liquid. The exhaust pressure at tailpipe is around 1 bar and it is common knowledge that water molar concentration is 14%. Therefore, the partial water vapour pressure can be deter-mined according to Dalton’s law which is 0.14 bar. The water vapour partial pressure specifies dew point, below which the water vapour condensates as shown in Figure 4, based on the data in [11]. At a pressure of 0.14 bar, the saturation temperature is 53 °C (see Figure 4).

In order to achieve a “closed-loop”-operation (e.g., on-board generation of water using exhaust gas) a system called WAHASY (WAter HArvesting SYstem) has been developed. Its primary target is to provide enough water in liquid state to match the required amount as needed for intended engine operation. This amount is given by Equation (2), where WFR stands for “Water to Fuel Ratio” (e.g., the volume of liquid water injected) compared to the volume of fuel and the WAHASY efficiency is the total efficiency of the system (e.g., the amount of water which can effectively be used for the water injection). In an ideally dimensioned system, this efficiency also matches the amount of water being condensed divided by the total amount of water present in the exhaust gas.

(2) $$\frac{WFR}{WC}=\Phi_{WAHASY}=\frac{M_{condensed_{water}}}{M_{water_{in_{exhaust_{gas}}}}}$$

**Figure 5:**
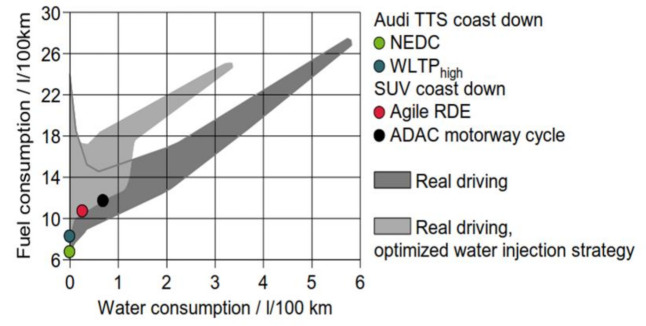
Water Consumption for Various Test Conditions [12].

Initial investigation in the past showed a wide array of water consumption figures when applying Water Injection, depending on test procedures and/or driving habits (see Figure 5).

Figure 5 shows that the required Water-to-fuel ratio (WFR)—even if it is able to raise up to 20%—is mostly under 10% in the tested drive cycles. This leads to a required WAHASY-efficiency of around 8% (up to 15% is considered for the most extreme “Real Drive” (RDE) profile). The efficiency of the WAHASY system is comprised of water condensation efficiency and the separation efficiency of small droplets from the exhaust stream.

## 4. Results

### 4.1. GT-Suite 1D Model

To determine the right WAHASY size, a GT-Suite model was developed and verified by engine testing. GT Suite is the industry-leading simulation tool with capabilities and libraries aimed at a wide variety of applications in automotive technology. Criteria of the decision matrix were:

- Limit system complexity
- Increase package compactness
- Maximize thermal performance
- Minimize heat dissipated through the LT coolant loop
- Minimize costs

**Figure 6:**
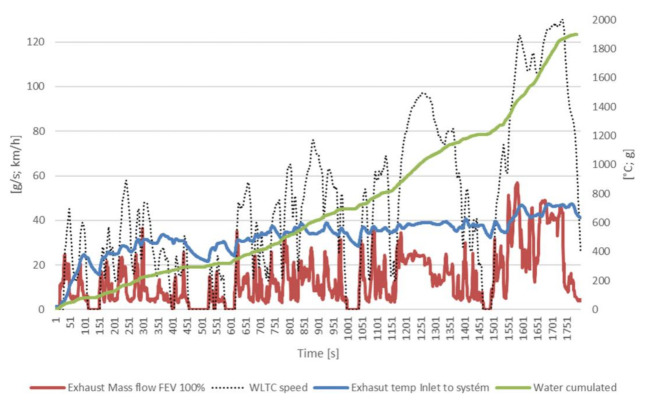
Data from the vehicle test.

A two-stage cooling design was selected as the best design (initial HT HEX followed by a second LT HEX) to condensate water vapour. A third device (“Harvester”) is intended to separate the condensate droplets from the exhaust gas flow. The GT-Suite 1D Tool was chosen to model behaviour measured on a real vehicle (see Figure 6). See the maximum available water content in exhaust gases below.

**Figure 7:**
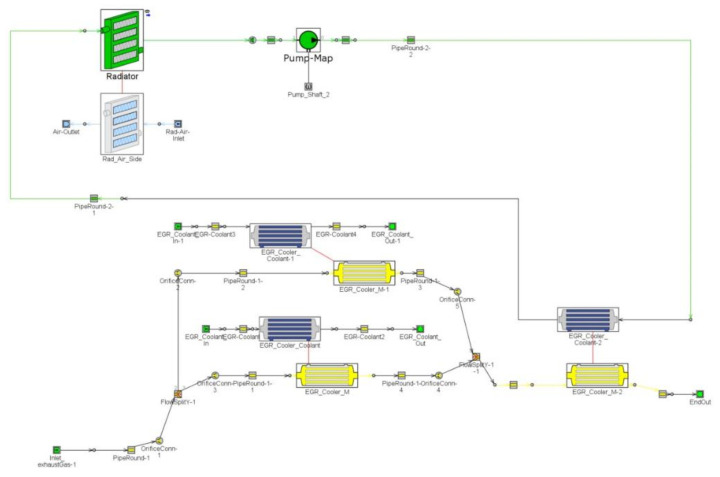
GT model of HAWASY system.

**Figure 8:**
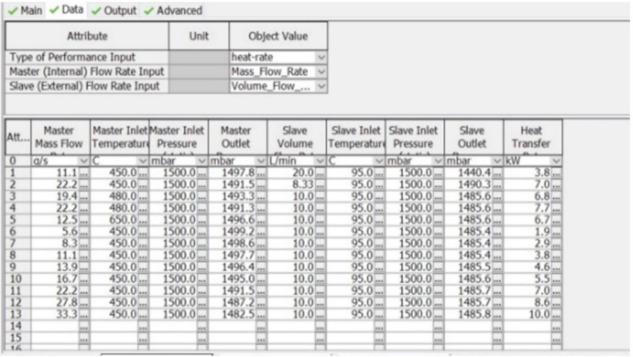
Thermal data of heat exchangers used for 1D simulation.

The data above will serve as inputs to the GT model (Figure 7), especially the inlet temperature and mass flow of exhaust. As mentioned above, the system has two coolant loops. High temperature (HT) and low temperature (LT). The high-temperature loop has two parallel coolers with a temperature of 90 °C. The low-temperature cooler is connected to a low-temperature radiator cooled by ambient air. The cooler thermal properties were taken from real calorimeter measurements (Figure 8).

**Figure 9:**
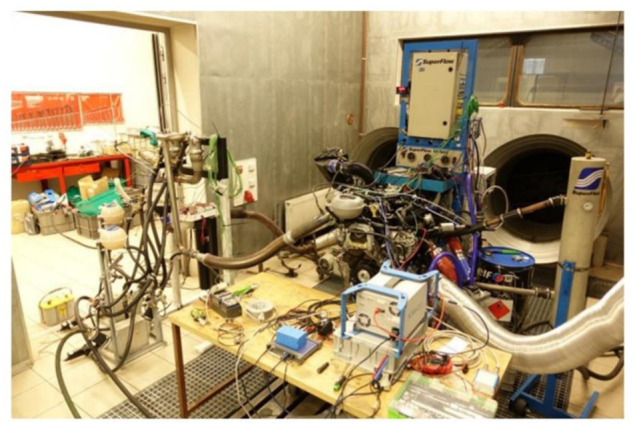
Engine test stand.

**Figure 10:**
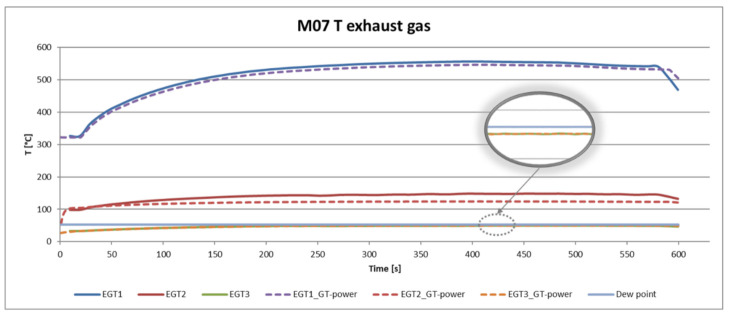
Comparison of exhaust temperatures between experiment and simulation.

To check proper function of the GT Suite model, an engine test was established (Figure 9), using the same engine as FEV their vehicle test (Figure 6). For repeatability reasons, stationary points from WLTC driving cycle measurements were selected. For M07 point the engine settings was 2700 rpm and torque 100 Nm which represents 31.4 g/s as exhaust mass flow. Exhaust gas temperature was monitored on the downstream and upstream of each cooler. Measured results data was used for comparison with the GT Suite model (Figure 10).

Figure 10 shows the three temperatures EGT1, EGT2 and EGT3 from the test. All of these sensors have a twin value from GT Suite. EGT1 is at the inlet temperature to the HT coolers, EGT2 temperature is inlet temperature to the LT cooler. EGT3 is the main temperature from the output of the LT cooler. It is obvious that this temperature is safely under the Dew point (53 °C) of water vapour in the exhaust calculated above.

**Figure 11:**
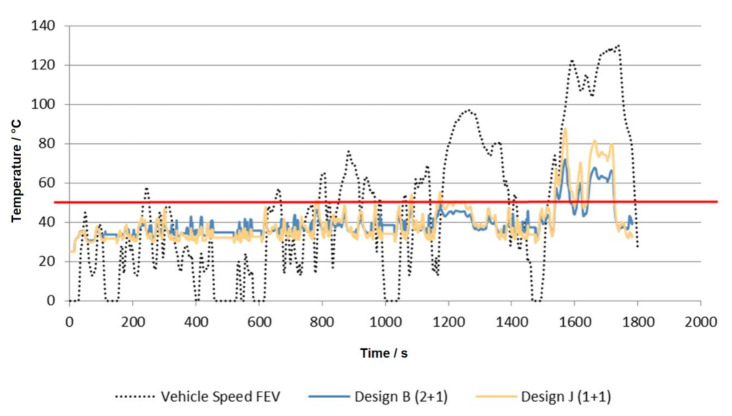
Comparison of different WAHASY designs.

The 1D model was verified by experiment and can be used for WAHASY modelling and finding its suitable parameters. Figure 5 shows that WAHASY with an efficiency of only 15% in worst conditions is necessary. Hypothetically, if applied to the WLTC cycle, only 300 mL of water is necessary to harvest. This would be enough to operate the WAHASY system and also to replenish the tank condensate. This is also the reason why the WAHASY is focused only on low load modes where lower back pressure losses in exhaust and better efficiency of condensate harvester are expected. Only 693 mL of water is available for the first 1000 s of WLTC cycles. By applying our GT-Suite model, it was found that just two coolers (one HT and one LT) are enough (Design—J). Figure 11 shows that up to the first 1000 s, both variants have similar efficiency. If the exhaust gas has more energy than our 1 + 1 design is able to cool, it will be automatically bypassed by the exhaust valve outside the WAHASY unit.

### 4.2. Harvester Separator Unit

**Figure 12:**
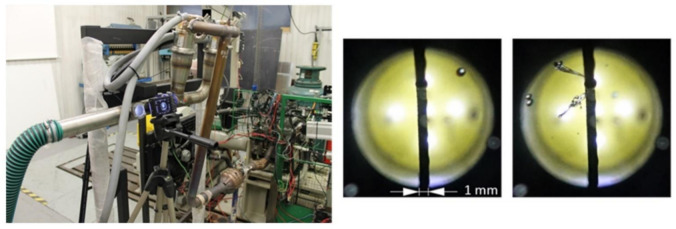
Condensate droplets size measuring.

As explained above, the efficiency of water vapour cooling under the dew point is only the first phase of total efficiency. The second phase concerns collecting the condensate droplets and separating them in the tank. For the unit to be developed, it was necessary to measure the size and distribution of droplets in the exhaust. An experiment was therefore carried out where photos were taken, through which droplet size and distribution could be measured indirectly (Figure 12).

**Figure 13:**
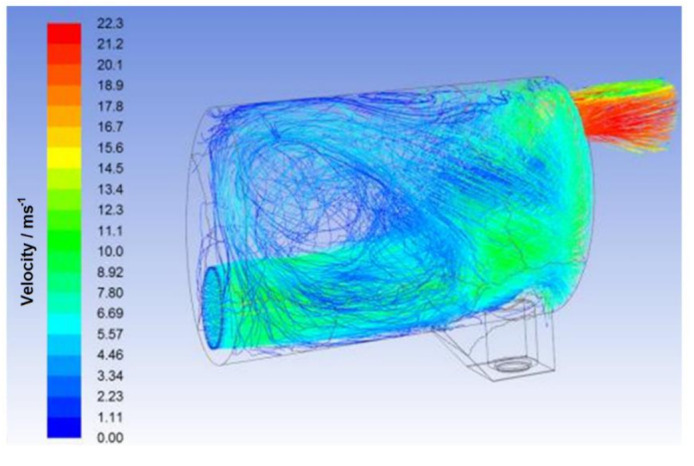
CFD Simulation of flow in harvester unit.

The mode of measured diameters was determined as 0.47 mm and the minimal diameter as 0.3 mm. In the CFD simulation, the diameter was set to uniform for all droplets with its value of 0.25 mm to overcome possible inaccuracy of measurements and simulate worse scenario. The CFD model analyses droplets movement by DPM (Discrete phase model) settings in Fluent (Figure 13). In DPM settings the interaction with continuous phase was enabled and the injection of water droplets was subjected to inlet surface. The diameter of droplets was assumed to be uniform with a value of 0.25 mm and mass flow rate of the droplets was set to 3.8 g/s, mass flow of total (water droplets and exhausts gas) was set to 47.9 g/s. This point comes from 88 s of the WLTC cycle considered aver-age value.

The calculation of harvester efficiency was determined as follows. If the water drop-lets touch the inside wall of the harvester, they are then considered “caught”. After calculation, the results of efficiency are at 95%.

### 4.3. Vehicle Experiment

**Figure 14:**
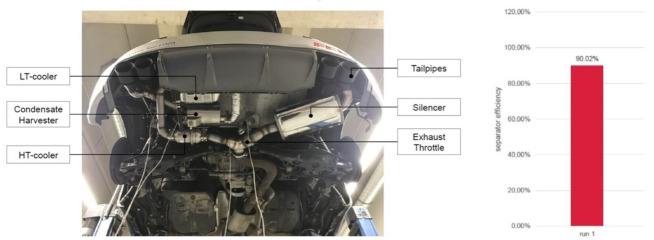
Vehicle test and vehicle test results.

After system simulation, a vehicle prototype was built to measure the actual efficiency of the WAHASY system. To simulate similar conditions, 88 s point of the WLTC cycle was simulated by driving at constant speed at 3rd gear and 3500 rpm to have identical inputs as during simulation. The results recorded in the graph (Figure 14) show that the run achieved an efficiency 90%.

## 5. Conclusions

The scope of the WAHASY project was to demonstrate the possibility of an autonomous, self-contained system, able to condensate and harvest sufficient amounts of water to allow a “maintenance-free” and “user-independent” water injection strategy.

WAHASY, a water condensation & harvesting system, was developed and subsequently proven through engine and vehicle testing. It has been demonstrated that sufficient water can be condensed and harvested. Analytical methods and simulation models have been worked up and a vehicle has been modified with the on-board WAHASY (FEV’s Audi TT-S WI Demonstrator Vehicle).

In the nearest future, additional tests allow extensive research of condensate. The comparison of the required condensation efficiency with the actual efficiency of this “first generation” WAHASY sample revealed the possibility to significantly reduce the size of the system without restricting its potential. Simplifying and downsizing the overall design will sup-port applications with different engine and exhaust system packages and lay-outs.

Tail pipe emissions have not been investigated during the initial study and will re-quire further attention. As demonstrated in another study, WI has a positive impact of NOx emissions but may create some increase of unburned HC [13]. This is especially a problem during the first 30 to 50 s after cold start, before the three-way catalyst achieves its light-off temperature. Another study [14] has indicated that a partial wash-out of un-burned HC can be achieved through water condensation. As water is not injected during cold start but WAHASY may be used, this could enable an emission advantage when using the unit.

Also, anti-freezing techniques must be investigated to make the system reliable in all weather conditions. Nevertheless, currently existing solutions for other fluids (e.g., as urea injecting) may be re-used if necessary.

Finally, self-contained water harvesting enables the option of wide-spreading on water injection as a future fuel consumption improvement technology without creating difficulties for final customers to accept. The possible positive global impacts of water injection applications on the environment and public health can also be documented by the following facts. Figure 3 shows that a gasoline engine with water injection can save more than 3% of fuel consumption. According to EUROPEAN VEHICLE MARKET STATISTICS, Pocketbook 2020/21 [15], 16.6 million new passenger cars were registered in the European Union in 2019, of which 60% with gasoline engines. If average emissions of 127 g CO_2_/km are considered, the application of water injection could save 531,495 ton of CO_2_ emission per year for new cars.

## Data Availability

Not Applicable.

## References

[1] Franzke B., Voßhall T., Adomeit P., Müller A. (2019). Water Injection for Meeting Future RDE Requirements for Turbocharged Gasoline Engines. MTZ Worldw..

[2] Hugon P. (1865). Improvement in Gas Engines. U.S. Patent.

[3] Water Injection More Power, Less Fuel Consumption. https://water-injection.fev.com/.

[4] Durst B., Unterweger G., Reulein C., Ruppert S., Linse D., Kern W. Increased performance of gasoline engines through various water injection concepts. Proceedings of the MTZ-Fachtagung Ladungswechsel im Verbrennungsmotor. 8.

[5] Pauer T., Frohnmaier M., Walther J., Schenk P., Hettinger A., Kampmann S. Optimization of gasoline engines through water injection. Proceedings of the 37th International Vienna Motor Symposium.

[6] Durst B., Landerl C., Poggel J., Schwarz C., Kleczka W., Hußmann B. BMW water injection: First experiences and future potential. Proceedings of the 38th International Vienna Motor Symposium.

[7] Hoppe F., Thewes M., Seibel J., Balazs A., Scharf J. (2017). Evaluation of the Potential of Water Injection for Gasoline Engines. Engines SAE Int. J. Engines.

[8] Hermann I., Glahn C., Kluin M., Paroll M., Gumprich W. Water Injection for Gasoline Engines—Quo Vadis?. Proceedings of the 5th International Conference Knocking in Gasoline Engines.

[9] Aqian L., Zhaolei Z., Tao P. (2020). Effect of water injection on the knock, combustion, and emissions of a direct injection gasoline engine. Fuel.

[10] Conway G. Injection of Alternative Fluids for Knock Mitigation. Proceedings of the International Powertrains, Fuels and Lubricants Meeting.

[11] The Engineering Toolbox: Water-Saturation Pressure. https://www.engineeringtoolbox.com/water-vapor-saturation-pressure-d_599.html.

[12] Thewes M. (2019). FEV Final Report. On-Board Water Generation from Exhaust for Water Injection in Gasoline Engines.

[13] Hunger M., Böcking T., Waither U., Günther M. Potential of Direct Water Injection to Reduce Knocking and Increase the Efficiency of Gasoline Engines. Proceedings of the 5th International Conference Knocking in Gasoline Engines.

[14] Rounds F.G., Bennett P.A., Nebel G.J. (2012). Some Effects of Engine-Fuel Variables on Exhaust Gas Hydrocarbon Content. J. Air Pollut. Control. Assoc..

[15] European Vehicle Market Statistics. http://eupocketbook.org/wp-content/uploads/2020/12/ICCT_Pocketbook_2020_Web.pdf.

